# Smac-mimetic enhances antitumor effect of standard chemotherapy in ovarian cancer models via Caspase 8-independent mechanism

**DOI:** 10.1038/s41420-021-00511-2

**Published:** 2021-06-04

**Authors:** Lidia F. Hernandez, Angie B. Dull, Soumya Korrapati, Christina M. Annunziata

**Affiliations:** 1 Women’s Malignancies Branch, Center for Cancer Research, National Cancer Institute, National Institutes of Health, Bethesda, MD USA; 2 Clinical Pharmacodynamics Program, Frederick National Laboratory/Leidos, Frederick, MD USA

**Keywords:** Ovarian cancer, DNA damage response

## Abstract

Ovarian cancer is the most lethal gynecological cancer in the US. Standard treatment consists of surgery followed by chemotherapies relying on apoptotic tumor cell death. Most women with advanced stage disease will relapse, suggesting that this disease is characterized by primary and acquired resistance to chemotherapy, and novel approaches to treatment are greatly needed. Low Caspase 8 expression levels in ovarian cancers correlate with resistance to apoptotic chemotherapy, and a subpopulation of patients with low Caspase 8 levels exhibit poorer overall survival after standard-of-care treatment. We hypothesized that low Caspase 8 function reduces the ability of cancer cells to undergo apoptosis when exposed to standard chemotherapy and that second mitochondria-derived activator of caspases (Smac)-mimetics could increase cell death in combination with chemotherapy. Here we show that combination treatment with a Smac-mimetic can target tumor cells with low Caspase 8 and induce necroptotic cell death. We investigated the in vitro effect of Smac-mimetic added to carboplatin and paclitaxel treatment of ovarian cancer cells expressing wild type and low Caspase 8 levels, which resulted in a 2–4-fold enhancement of cell death. Mice bearing subcutaneous or intraperitoneal ovarian xenografts showed greater aggressiveness of Caspase 8-deficient versus wild-type tumors; combined in vivo treatment with chemotherapy and Smac-mimetic resulted in >50% decrease in low Caspase 8 xenograft growth, as well as significantly enhanced overall survival, especially when given simultaneously with paclitaxel. Surprisingly, Smac-mimetic on the same day as carboplatin decreased mouse survival compared to when it was given on a sequential day of treatment. The antagonism was associated with a decrease in DNA damage markers, emphasizing the importance of optimizing timing of drug administration. Clinical validation of such approaches is needed to increase the effectiveness of current standard ovarian cancer treatment.

## Introduction

Ovarian cancer is expected to produce 21,750 new cases and 13,940 deaths in the US in 2020^1^. Since the introduction of platinum/taxane therapies in the 1990s, there have been few improvements in standard chemotherapy regimens especially for women with homologous recombination-proficient cancers, and 5-year survival for advanced disease remains at 47% (http://seer.cancer.gov/statfacts/html/ovary.html). Standard treatments typically rely on apoptotic cell death for targeting tumor cells, an effective primary treatment, but most women relapse and exhibit acquired resistance to chemotherapy, highlighting the need for secondary lines of treatment in order to improve outcome.

Recent molecular characterization of ovarian cancers has identified subgroups, adding prognostic value as well as informing new therapeutic approaches^2,3^. We recently established the role of nuclear factor (NF)-κB on inflammation and tumor cell survival in ovarian cancer^4^ and demonstrated the dual role of the pro-apoptotic protein Caspase 8 in ovarian cancer. These studies have shown that patient subgroups expressing high Caspase 8 and high NFκB activity have longer overall survival, but low Caspase 8 and low NFκB activity corresponded with shortest overall survival^5^. We also showed that ovarian cancers depleted of Caspase 8 can be susceptible to cell death by caspase-independent necroptosis^5^. In the current studies, we hypothesized that low Caspase 8 and low NFκB activity can limit apoptosis and decrease the benefit of apoptotic cell death after conventional chemotherapy. We investigated the effect of combined chemotherapy and Smac-mimetic treatment as inducers of both apoptotic and necroptotic cell death. In vitro and in vivo, we found ovarian cancer cell death to be similarly pronounced in both Caspase 8 high-expressing and low-expressing ovarian cancers after combined treatment, suggesting that survival benefits may be possible with this therapeutic approach.

## Results

### Low Caspase 8 suggests resistance to chemotherapy

**Fig. 1:**
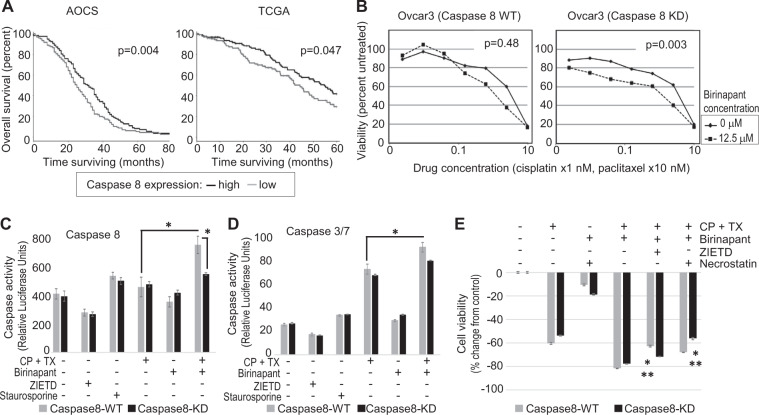
Low Caspase 8 increases sensitivity of ovarian cancer cell lines to combined chemotherapy treatments. **A** Overall survival in patients with high (above the median) or low (below the median) Caspase 8 in two datasets shows that low Caspase 8 consistently relates to shorter overall survival. **B** Ovcar3 cells transduced with either control shRNA (Caspase 8 WT) or Caspase 8 shRNA (Caspase 8 KD) were treated with increasing concentrations of combined cisplatin and paclitaxel in the presence or absence of birinapant (12.5 μM) for 72 h. Cell viability was measured by XTT assay. Representative results are expressed as average ± S.E.M., *n* = 4 and shown as percentage of change from vehicle control. **C** Ovcar3 cells expressing either control (Caspase 8 WT) or Caspase 8 shRNA (Caspase 8 KD) were exposed to combined cisplatin (2.5 µM) and paclitaxel (25 nM) and/or Caspase 8 inhibitor ZIETD (25 µM), birinapant (12.5 μM), and staurosporine (1 µM) for 18 h to induce caspase activity. Results of Caspase 8 cell-based assay are expressed as average luciferase units ± S.E.M., *n* = 4. Single asterisk (*) represents *p* < 0.05 based on *t* test. **D** Caspase 3 and 7 activity was measured in the described cell lines and exposed to similar conditions described in **C**. **E** Ovcar3 cells expressing either control (caspase 8 WT) or Caspase 8 shRNA (Caspase 8 KD) were exposed for 72 h to combined cisplatin (2.5 µM) and paclitaxel (25 nM), birinapant, 12.5 μM, and/or ZIETD (25 µM) or NEC1 (25 µM) as indicated. Viability was assessed by XTT assay. Results are expressed as average ± S.E.M., *n* = 3. Single asterisk (*) represents *p* < 0.01 based on *t* test comparing Caspase 8 WT versus KD; Double asterisks (**) represent *p* < 0.01 based on *t* test comparing ZIETD to Necrostatin.

In two large genomic analyses, low Caspase 8 indicated shorter overall survival (Fig. 1A). The Australian Ovarian Cancer Study (AOCS) contains gene expression data from 267 cases, 87% of which were stage III or IV, and 92% were serous subtype^2^; The Cancer Genome Atlas (TCGA) profiled 489 cases that were stage II–IV and all were high-grade serous cancers^3^. All patients in both datasets received platinum-based chemotherapy for up-front treatment after tumors were harvested for molecular analyses. In the AOCS and TCGA datasets, expression of Caspase 8 below the median was associated with worse outcome compared to cases with Caspase 8 expression above the median (*p* < 0.05). This suggested poor response to chemotherapy related to lack of Caspase 8 activity. We sought to improve response to chemotherapy in cell lines representative of low Caspase 8 activity. We therefore tested the pair of Ovcar3 cells that we had established, with high endogenous Caspase 8 (wild type (WT)) or stable knockdown of Caspase 8 (KD). The matched Ovcar3 cell lines are NFκB-dependent, based on previously published work^4^, and we sought to increase chemotherapy sensitivity by invoking cell death independent of Caspase 8. The SMAC-mimetic birinapant has been shown to induce receptor-interacting protein kinase (RIPK)1-dependent necroptosis^6^. We hypothesized that depletion of cIAP1 with the SMAC-mimetic birinapant would increase the availability of uncleaved RIPK1^7^ and further enhance the effect of Caspase 8 depletion. Interestingly, the addition of birinapant to cisplatin and paclitaxel had little effect on Caspase 8-high cells but increased the sensitivity of Caspase 8-deficient cells especially at low concentrations of chemotherapy (Fig. 1B, *p* < 0.01). These results suggest a mechanism of either intrinsic apoptosis or necroptosis that bypasses Caspase 8.

### Caspase 8 activity mediates apoptotic cell death of ovarian cancer cells

We have previously shown that tumor necrosis factor-alpha (TNFα) can promote cell proliferation when cIAP1 is present, as well as induce apoptosis when it is absent^5^. Caspase 8 is involved in this complex mechanism mediating pro-survival NFκB signaling or triggering extrinsic apoptosis downstream of short-term stimulation with TNFα in ovarian cancer cells (Supplementary Fig. 1). We proceeded to investigate whether the apoptotic mechanisms were activated in cells treated with chemotherapy. In this short-term assay, Ovcar3 cells were transduced with Caspase 8 shRNA or control shRNA in order to measure the input of Caspase 8 in chemotherapy-induced cell death signaling. Interestingly, depletion of cIAP1 with the addition of SMAC-mimetic birinapant to the combination chemotherapy did activate Caspase 8 in Ovcar3 cells expressing Caspase 8, and this rise was attenuated by Caspase 8 depletion (Fig. 1C, *p < 0.05). Assay of Caspase3/7 activity demonstrated rise with cisplatin and paclitaxel, suggesting that these drugs activate intrinsic apoptosis signaling (Fig. 1D). Caspase3/7 activity was augmented with the addition of birinapant (*p < 0.05) and was not significantly different in cells with Caspase 8 knocked down (*p* = 0.16). Since Caspase 8-low cells were more susceptible to killing with the combination of chemotherapy and birinapant (Fig. 1B), we asked whether apoptosis or necroptosis contributed to the mechanism of cell death. Cells were treated with the combination of cisplatin, paclitaxel, and birinapant. In Ovcar3 cells with high Caspase 8, inhibition of apoptosis with ZIETD provided relatively more protection than inhibition of necroptosis with necrostatin (Fig. 1E). This effect was reversed in Ovcar3 cells with low Caspase 8, suggesting that cells with low Caspase 8 activity are more likely to die by necroptosis (Fig. 1E, *p < 0.01 comparing Caspase 8 WT versus KD; **p < 0.01 comparing ZIETD to Necrostatin). Changes in apoptosis markers cleaved caspase 3 and cleaved poly (ADP-ribose) polymerase (PARP) were assessed by western blot using similar treatment conditions; necroptosis proteins, phosphorylated MLKL, and phosphorylated RIP3 were also measured. Blocking apoptosis with ZIETD again had a more prominent effect in the Caspase 8-WT cells, and inhibition of necroptosis with necrostatin had a greater effect on cell death markers in Caspase 8-KD cells (Supplementary Fig. 2B, C).

### Smac-mimetic birinapant combined with carboplatin and paclitaxel increases cell death in NFκB-independent Caspase 8-high and Caspase 8-low cells

**Fig. 2:**
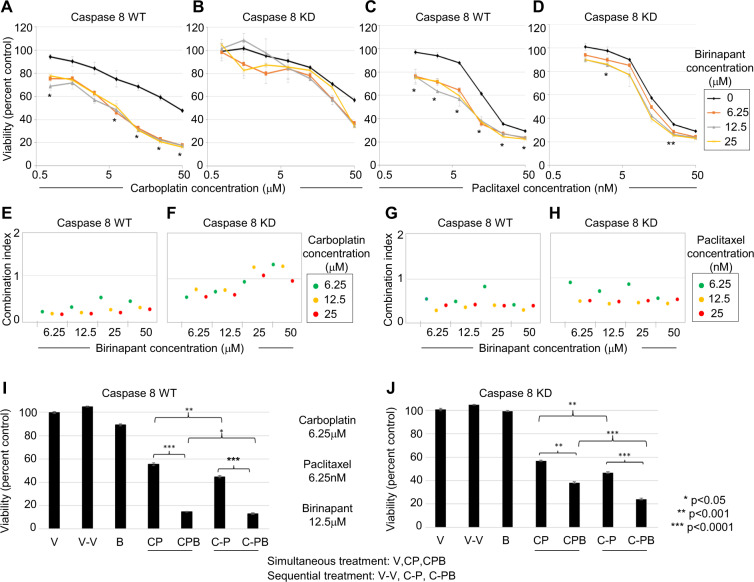
Smac-mimetic birinapant synergizes with paclitaxel and antagonizes carboplatin in Caspase 8-depleted Ovcar8 cells in vitro. Ovcar8 cells transduced with control shRNA (Caspase 8 WT) (**A**) or Caspase 8 shRNA (Caspase 8 KD) (**B**), respectively, were simultaneously treated with varying concentrations of carboplatin and birinapant for 72 h. Cell viability was measured by XTT assay. Results are expressed as average ± S.E.M., *n* = 3, and shown as percentage of change from vehicle control; single asterisk (*) represents *p* < 0.05, double asterisks (**) *p* < 0.01 of 25 µM Birinapant versus carboplatin, based on *t* test. Ovcar8 cells transduced with either control shRNA (Caspase 8 WT) (**C**) or Caspase 8 shRNA (Caspase 8 KD) (**D**) were treated with varying simultaneous concentrations of paclitaxel and birinapant for 72 h, and cell viability was assessed as described above. **E**–**H** Combination indexes for the matched Ovcar8 cell lines at 6.25 µM (green) 12.5 µM (yellow), and 25 µM birinapant^2^ concentrations were analyzed by Compusyn and indicated on graphs. **I**, **J** Ovcar8 cells transduced with either control shRNA or Caspase 8 shRNA were simultaneously or sequentially treated with 6.25 µM carboplatin, 6.25 nM paclitaxel, and 12.5 µM birinapant for 72 h. Cell viability was measured by XTT assay as described. Results are expressed as average ± S.E.M., *n* = 6, and shown as percentage of change from vehicle control. UT untreated, V drug vehicle alone, B birinapant, CP carboplatin/paclitaxel, CPB carboplatin/paclitaxel/birinapant, V-V drug vehicle 24 h + drug vehicle 48 h, C-PB carboplatin 24 h + paclitaxel/birinapant 48 h, C-P carboplatin 24 h + paclitaxel 48 h. Single asterisk (*) represents *p* < 0.05, double asterisks (**) *p* < 0.001, triple asterisks (***) *p* < 0.0001 based on *t* test for the indicated comparisons, shown as percentage of change from untreated.

Ovcar3 cells (NFκB dependent) and Ovcar8 cells (NFκB independent) express similar levels of endogenous Caspase 8^5^. In order to establish whether the benefit of birinapant addition to chemotherapy was related to NFκB dependence, we investigated whether the patterns of sensitivity to chemotherapy and Caspase 8-independent cell death we observed in Ovcar3 cells would also occur in Ovcar8 cells. We produced a pair of Ovcar8 cells, with high endogenous Caspase 8 or stable knockdown of Caspase 8 (Supplementary Fig. 2). The matched Ovcar8 cell lines showed unique pattern of sensitivity to the combination of carboplatin and paclitaxel; Caspase 8-low Ovcar8 cells were more resistant to carboplatin (higher IC50) than Caspase 8-high cells (Fig. 2A, B). Addition of birinapant to carboplatin significantly increased sensitivity of Caspase 8-high cells over a large range of concentrations (Fig. 2A) but did not similarly affect Caspase 8-low cells (Fig. 2B). Treatment of Caspase 8-high and Caspase 8-low cells with paclitaxel yielded comparable sensitivities, and addition of birinapant increased paclitaxel sensitivity of both cell types at the indicated concentrations (Fig. 2C, D). The results suggest that both NFκB-dependent and NFκB-independent ovarian cancer cells can be targeted for additional cell death by chemotherapy and birinapant combined treatment, though the magnitude of effect may differ based on Caspase 8 expression.

### Smac-mimetic birinapant synergizes with paclitaxel and antagonizes carboplatin in Caspase 8-depleted cells

In order to better assess whether a combination treatment of birinapant with carboplatin and paclitaxel together would yield a mathematically synergistic effect, we analyzed the combination indexes (CIs)^7^ of the paired set of Ovcar8 cells treated simultaneously with carboplatin/birinapant or paclitaxel/birinapant. Caspase 8-high cells showed synergism with a wide dose range of birinapant, combined with each carboplatin and paclitaxel, as indicated by a CI < 1. Caspase 8-low cells treated with carboplatin, however, showed moderate antagonism of birinapant at high concentrations and mild synergism at lower concentrations. When combined with paclitaxel, birinapant was synergistic over a wide range of concentrations (Fig. 2E–H). These data suggest that in order to achieve the most beneficial synergism between the combination of all three drugs, low concentrations of chemotherapeutic agents as well as birinapant should be used in vitro. Additionally, sequential treatment of paclitaxel–birinapant after carboplatin dosing would avoid any antagonistic effects.

### Sequential combination treatment of birinapant and chemotherapy provides maximum cell death benefit to both Caspase-high and Caspase-low cells in vitro

Caspase 8-high and Caspase 8-low cells were exposed to carboplatin, paclitaxel, and low birinapant in synergistic combinations. In order to assure optimal synergistic conditions, simultaneous treatment with the three drugs was compared to sequential treatment, consisting of a 24-h treatment with carboplatin, followed by paclitaxel or paclitaxel–birinapant combination, as described. Caspase 8-high cells significantly benefited from the combined effect of birinapant on chemotherapy, with a 3–4-fold increase in cell death; sequential treatment decreased the level of viability from 15% following simultaneous treatment to 13% following sequential treatment (Fig. 2I, *p < 0.05). Caspase 8-low cells also showed a moderate but significant 1.5-fold enhanced cell death with simultaneous birinapant combined with chemotherapy. Importantly, in these cells with low Caspase 8, sequential treatment significantly enhanced cell death compared to simultaneous exposure to the three drugs, from 38% viability with simultaneous treatment to 24% viability with sequential treatment (Fig. 2J, ***p < 0.001). These results are consistent with the observed antagonism between carboplatin and birinapant and further support the idea that maximum benefit may come from sequential treatment of birinapant and paclitaxel to carboplatin-exposed ovarian cancer cell lines.

### Caspase 8-low xenografts grow aggressively, and mice receiving in vivo sequential combination treatment with birinapant and chemotherapy benefit from a delayed rate of tumor growth

**Fig. 3:**
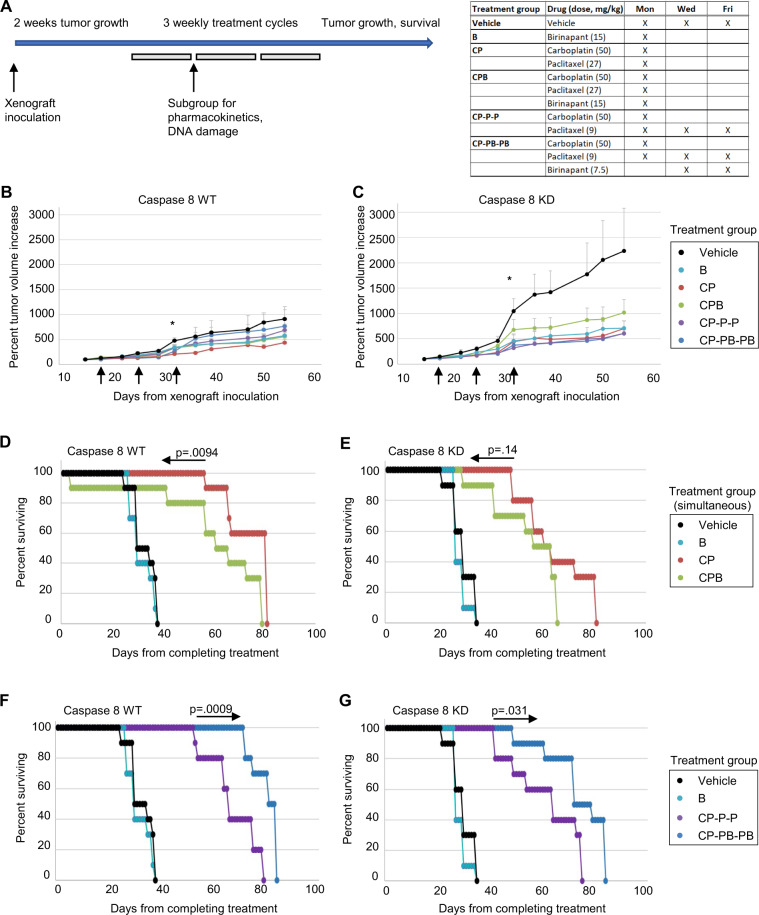
In vivo combined chemotherapy–birinapant treatments reduces xenograft growth rate and overcomes carboplatin antagonism. **A** Dose and administration schedule for carboplatin, paclitaxel, and birinapant. Mice were inoculated with human ovarian cell lines Ovcar8 with Caspase 8 control (WT) or knockdown (KD). When subcutaneous tumors reached 100–200 mm^3^ or at 15–20 days for post intraperitoneal inoculations, mice were randomized into groups of 5. Aqueous solutions of birinapant (Tetralogic, in citrate buffer), carboplatin (Teva), and paclitaxel (Teva) were administered intraperitoneally in 500 µl volumes once weekly × 3 weeks or thrice weekly × 3 weeks at the indicated doses. For simultaneous treatments, drug combinations were administered at the same time (CP, CPB), while for sequential treatments, drugs were administered simultaneously on days 1, 3 and 5 (CP-P-P; CP-PB-PB) at doses indicated in the chart. Subcutaneous tumors were measured twice a week as described for 50–60 days or when tumors reached humane endpoints, and IP tumors were assessed after 70–80 days or when health assessments indicated that a humane endpoint had been reached. **B** Subcutaneous tumor volume increases during and following chemotherapy/birinapant combination treatments of Ovcar8 xenografts expressing wild-type Caspase 8. Points are average of 5 mice; weekly treatments are indicated by black arrows. **C** Subcutaneous tumor volume increases during and following chemotherapy/birinapant combination treatments of Ovcar8 xenografts expressing low Caspase 8. Points are average of 5 mice; weekly treatments are indicated by black arrows. Single asterisk (*) represents *p* < 0.05, based on *t* test, and shown as percentage of change from vehicle-treated mice. **D**–**G** Overall survival of mice bearing intraperitoneal tumors expressing control (Caspase 8 WT) or low Caspase 8 (Caspase 8 KD), treated with simultaneous (**D**, **E**) or sequential (**F**, **G**) chemotherapy/birinapant administration as described in **A**. *p* Values represent two-tail, log-rank comparisons between the indicated survival curves.

We developed two approaches using our in vitro studies to inform in vivo studies of cooperative effects of birinapant and chemotherapy treatments (Fig. 3A). In a subcutaneous tumor growth model, mice were inoculated with either high Caspase 8 or Caspase 8-depleted cells in order to induce palpable tumors. After 2–3 weeks, mice were randomized and treated with either simultaneous doses of carboplatin, paclitaxel, and birinapant or sequential paclitaxel–birinapant treatments following carboplatin (Fig. 3A). Subcutaneous Caspase 8-deficient xenografts were much more aggressive than high-Caspase 8 tumors in total growth (Fig. 3B, C), consistent with the patient data showing shortest overall survival in NFκB-low, Caspase 8-low cases^5^. Chemotherapy treatment with or without birinapant decreased tumor growth rate of high-Caspase 8 xenografts by 1/3 to 1/2 after 8 weeks (Fig. 3B, *p < 0.05 compared to vehicle). Chemotherapy–birinapant combinations were most effective in reducing Caspase 8-deficient xenografts tumor growth rate by 2/3 after 8 weeks (Fig. 3C, *p < 0.05 compared to vehicle). Interestingly, simultaneous chemotherapy–birinapant treatment appeared to attenuate tumor reduction, suggesting an in vivo antagonistic effect of birinapant, which was avoided by the sequential dosing schedule.

In separate experiments, we used an orthotopic intraperitoneal inoculation model to mimic advanced-stage ovarian cancer. This model allowed us to investigate whether the above-described effects of treatments would affect overall survival of mice in a model of metastatic, disseminated abdominal tumors. With vehicle treatment, mice bearing Caspase 8-low tumors showed significantly shorter overall survival compared to their Caspase 8-high counterparts, consistent with the increased growth of the Caspase 8-low tumors (*p* < 0.05). High-Caspase 8 xenograft-bearing mice had a 20–30 day improvement on overall survival after treatment with chemotherapy + /− birinapant (Fig. 3D, F). Caspase 8-depleted xenograft-bearing mice showed >30 days improvement in overall survival when administered chemotherapy + /− birinapant (Fig. 3E, G). Notably, simultaneous treatment of carboplatin and birinapant failed to improve response to chemotherapy alone and significantly reduced the benefit of chemotherapy in Caspase 8-high cells, suggesting an antagonistic effect of birinapant on carboplatin when administered simultaneously in vivo (Fig. 3D, E). In contrast, when carboplatin was administered on day 1 and birinapant was given on days 3 and 5 with paclitaxel treatment, mice with either xenograft experienced significantly longer overall survival than without birinapant (Fig. 3F, G). Again, this highlights consistent synergy of birinapant with paclitaxel but antagonism with carboplatin.

### NFκB activity does not follow patterns of birinapant chemo-sensitization in Caspase 8-high or Caspase 8-low cells

**Fig. 4:**
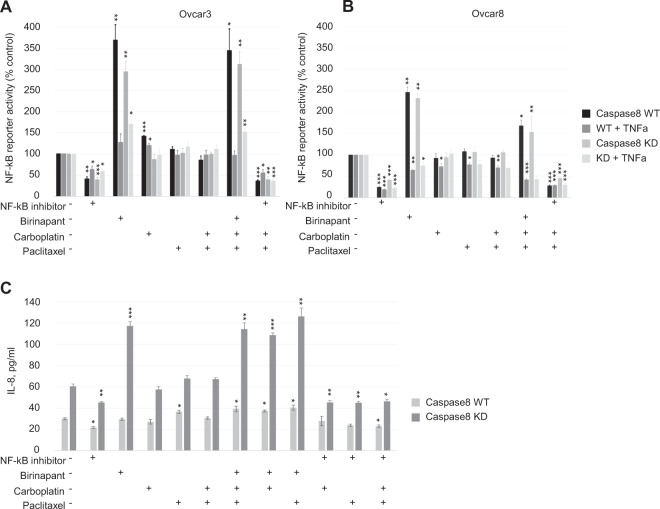
In vitro birinapant treatment inhibits TNFα-induced NFκB activity in NFκB-independent ovarian cancer cells. **A**, **B** NFκB signaling in each Caspase 8 WT and Caspase 8 KD Ovcar3 (**A**) and Ovcar8 (**B**) cell lines expressing a reporter was measured with luminescence. Cells were treated with simultaneous combinations of carboplatin, paclitaxel, birinapant, and IKKβ inhibitor IV for 18 h as indicated, and TNFα (10 ng/ml) added for the last hour. Data are arbitrary luciferase units normalized to viable cell number, *n* = 8, expressed as percentage of vehicle control. **C** IL-8 cytokine levels in Ovcar8 Caspase 8 WT and Caspase 8 KD cell lines after treatment with chemotherapy, birinapant, or IKKβ inhibitor IV for 18 h, as described, *n* = 8. Values are pg/ml.

Ovcar3 and Ovcar8 paired cell sets with and without Caspase 8 were studied to assess NFκB activity under synergistic conditions of combined treatments. We investigated the effect of single and combined drug treatments on Caspase 8-high and Caspase 8-low cells transiently expressing an NFκB luciferase reporter construct in the presence or absence of TNFα (Fig. 4). NFκB-dependent Ovcar3 cells with Caspase 8-high and Caspase 8-low expression showed, respectively, 3.5- and 2.5-fold increased NFκB activity after birinapant single or combined treatment under unstimulated conditions. Under stimulation with TNFα, birinapant did not further increase NFκB activity in Caspase 8-high cells compared to TNFα stimulation alone, and NFκB activity was 1.5-fold increased in caspase 8-low cells, likely due to the known role of Caspase 8 in classical NFκB signaling in NFκB-dependent ovarian cells (Fig. 4A). In NFκB-independent Ovcar8 cells with Caspase 8-high and Caspase 8-low, birinapant single and combination treatment resulted in an attenuated but similar 2.3- and 2.4-fold increased activity in steady-state cells, but in a 1.5- and 2.0-fold decreased activities, respectively, in the presence of TNFα, compared to TNFα stimulation alone (Fig. 4B). IL-8 secreted level was measured in Ovcar8 cells as a reflection of NFκB functional activity without TNFα stimulation (Fig. 4C). Unexpectedly, IL-8 levels were higher in Caspase 8-depleted Ovcar8 cells compared to control cells. Birinapant treatment further increased levels of this NFκB target gene product in Caspase 8-low cells alone and in combination with carboplatin, paclitaxel, or both drugs. Taken together, these experiments suggest that NFκB activity is unlikely to influence the synergy or antagonism of birinapant with paclitaxel or carboplatin, as no clear distinctions between treatments or cell lines occurred in these assays.

### Antagonistic high-dose birinapant combined with chemotherapy lowers Caspase 9 activity in Ovcar8 cells, suggesting attenuation of intrinsic apoptotic cell death

**Fig. 5:**
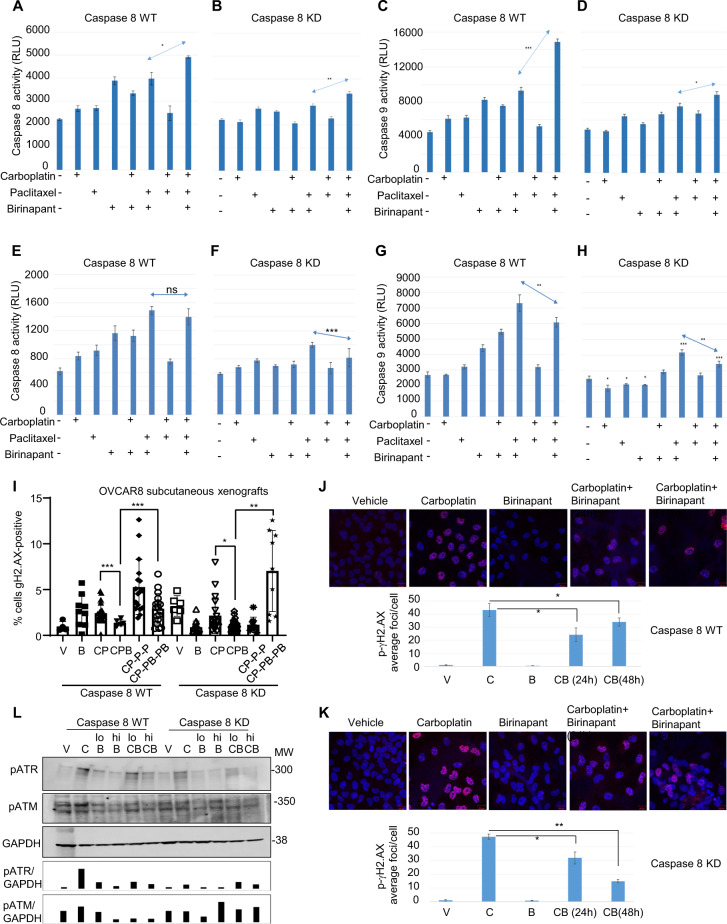
Caspase activity response to birinapant/chemotherapy treatment is attenuated by simultaneous administration of carboplatin. Caspase 8 (**A**, **B**) and Caspase 9 (**C**, **D**) activity was measured after treatment of Caspase 8 WT and Caspase 8 KD cells with carboplatin (6.25 µM), paclitaxel (6.25 µM), and low Birinapant (12.5 µM) for 18 h. Values are arbitrary Caspase units, *n* = 8. Single asterisk (*) represents *p* < 0.05, double asterisks (**) *p* < 0.001, triple asterisks (***) *p* < 0.0001 based on *t* test. **E**–**H** Similar experiments were carried out using high Birinapant (25 μM). **I** Percentage of γH2A.X-positive cells were measured by immunofluorescence in subcutaneous tumors from mice treated as described in Fig. 3. *p < 0.05; **p < 0.01; ***p < 0.001. **J**, **K** γH2AX response was measured in Caspase 8 WT (**J**) and Caspase 8 KD (**K**) Ovcar8 cells treated with carboplatin (6.25 µM) and/or high birinapant (25 µM) simultaneously for 24–48 h as indicated, using the Zeiss Zen Software from NCI Confocal Core Facility. Data are average γH2A.X foci per cell, *n* = 100. Single asterisk (*) represents *p* < 0.05, double asterisks (**) *p* < 0.01, based on *t* test. **L** Western blot analysis of total protein lysates after 18 h single or simultaneous treatment with carboplatin (C, 6.25 µM), birinapant (lo B, 12.5 µM or hi B, 25 µM). Loading amounts were assessed by GAPDH staining and ratios of protein to loading control were calculated using ImageJ.

NFκB-independent Ovcar8 Caspase 8-high and Caspase 8-low cells were treated with simultaneous combinations of carboplatin, paclitaxel, and low (synergistic) or high (antagonistic) birinapant concentrations. Low birinapant concentration increased caspase activity with each single drug as well as combined, in cells expressing high levels of Caspase 8 (Fig. 5A, C); the triple drug combination induced the highest levels of Caspase 8 and Caspase 9 activity, supportive of cell death by extrinsic as well as intrinsic apoptosis. Caspase 8-low cells (Fig. 5B, D) only showed enhancement of caspase activities in paclitaxel single drug or in combination, indicating that triple drug combination was beneficial—albeit to a lesser degree, combining to induce cell death in these chemotherapy-resistant cells.

Under high, antagonistic birinapant concentrations, Caspase 8-high cells showed caspase activity induction by single and double combinations, but triple drug combinations did not increase Caspase 8 activity (Fig. 5E) and decreased Caspase 9 activity (Fig. 5G) compared to the combination of birinapant and paclitaxel. Caspase 8-low cells showed minimal significant benefit of double drug combinations and also a decrease in caspase activities under triple combinations (Fig. 5F, H). Taken together, these experiments provide molecular evidence that birinapant synergizes well with paclitaxel under a range of concentrations in cells expressing high and low Caspase 8 levels, while in the presence of carboplatin, only low birinapant concentrations induce increased caspase activities, while high birinapant with carboplatin is detrimental.

### Simultaneous birinapant treatment interferes with carboplatin-induced DNA damage

Carboplatin causes DNA interstrand crosslinks, which lead to single-strand breaks that can be recognized by DNA damage response pathways. Phosphorylated (p)-ATR and p-ATM proteins are recruited to strand break sites and phosphorylate γH2A.X. We asked whether birinapant interfered with carboplatin DNA damage response. Subcutaneous xenografts were obtained after 1 week of in vivo treatment with simultaneous or sequential doses of birinapant and chemotherapy, as described (schema in Fig. 3A). γH2A.X staining indicative of DNA damage response was scored. Higher amounts of γH2A.X were present in both Caspase 8-high and Caspase 8-low tumors from mice treated with sequential birinapant–paclitaxel treatment after carboplatin but not simultaneous treatment (Fig. 5I, *p < 0.01). This finding is consistent with our in vivo data showing that the simultaneous presence of birinapant with carboplatin inhibits its antitumor function. These results suggest that birinapant blocks the ability of carboplatin to induce DNA damage, as measured by γH2A.X foci. The presence of birinapant significantly reduced γH2A.X foci in both Caspase 8-high and Caspase 8-low xenografts. The simultaneous administration of birinapant following carboplatin, and with paclitaxel, significantly increased DNA damage compared to the simultaneous administration.

In a separate experiment, Caspase 8-high and Caspase 8-low Ovcar8 cells were treated in vitro with simultaneous combination doses of low and high birinapant and carboplatin for 24 h and fixed as described. p-γH2AX staining (Fig. 5J, K) showed a significant increase in response to single carboplatin treatment and a significant attenuation when administered together with a high dose of birinapant. Based on the kinetics of the measurable changes in a culture of unsynchronized cells, the 24-h timepoint is the most relevant to measure the changes in gamma-H2A.X phosphorylation. The 48-h timepoint was included to show that the effect persists. Levels of pATR and pATM increased in response to single carboplatin treatment of Caspase 8-high and Caspase 8-low Ovcar8 cells, indicating DNA damage response (Fig. 5L). Conversely, these proteins decreased when high-dose birinapant was administered simultaneously with carboplatin. These experiments support the idea that high-dose birinapant administered simultaneously with carboplatin prevents carboplatin-induced DNA damage response as measured by a decrease in phosphorylation and activation of both ATM and ATR proteins and decreased γH2A.X accumulation in DNA. These effects likely attenuate the ability of carboplatin to kill ovarian cancer cells.

## Discussion

Ovarian cancer is recognized to be an extremely heterogeneous disease, presenting unique treatment challenges, and successful therapeutic approaches are increasingly guided by specific genetic and somatic molecular profiles of patient subpopulations in order to maximize clinical benefits. We previously demonstrated that NFκB inhibition can induce cell death by apoptosis in ovarian cancer cells dependent on a TNFα-induced pathway for proliferation^4^. More recently, we have shown that Caspase 8 plays a dual role in ovarian cancer, regulating apoptosis and necroptosis downstream of TNFαR1^5^.

In the current study, we show that ovarian cancers expressing low levels of Caspase 8 may be resistant to standard apoptotic-inducing chemotherapy treatments, because they exhibit poorer prognosis than ovarian cancers expressing high Caspase 8 levels. We hypothesized that such patient populations could be targeted by additional SMAC-mimetic treatment in order to deplete cIAP1 and enhance cell death by necroptosis. Using representative ovarian cancer cell lines, we show that the SMAC-mimetic birinapant combined with chemotherapy increased cell death in cells with low Caspase 8, representing the poor prognostic subgroup. Further, in vitro combination treatment with carboplatin, paclitaxel, and low concentration of SMAC-mimetic resulted in synergistic effects that were greater when SMAC-mimetic is administered sequentially, instead of simultaneously, with carboplatin in both high and low Caspase 8 ovarian cancer cells. Using these cell lines to induce xenograft tumors in nude mice, we show that low Caspase 8 cells induced more rapidly growing subcutaneous tumors and that in vivo combination treatments reduced both Caspase 8-high and Caspase 8-low tumor growth. The addition of birinapant in the subcutaneous tumor mouse model did not significantly change tumor volume, as all the treatments kept the tumors small in this 8-week timeframe. There are several reasons why the subcutaneous model does not directly mimic the in vitro culture results. Drug delivery to culture is more uniform and likely reaches higher levels when placed directly onto the cells; metabolism of drugs in culture is likely different than metabolism in mouse circulation between peritoneal cavity and subcutaneous capillaries; the mouse subcutaneous tissue microenvironment may also contain other signaling factors that provide chemoresistance, such as cytokines and adhesion molecules.

Long-term survival studies in the orthotopic intraperitoneal mouse model showed significant overall survival benefit with the sequential birinapant treatment in mice bearing either the Caspase 8-high or the Caspase 8-low tumors. Importantly, in this model, the survival of the mice bearing the more aggressive Caspase 8-low ovarian cancer cells reached that of the mice with the Caspase 8-high tumors. This suggests that the addition of birinapant to chemotherapy could potentially benefit women with aggressive ovarian cancer if it is added in the correct sequence.

Surprisingly, the presence or absence of NFκB activity did not affect the pattern of cells’ response to the combinations of carboplatin, paclitaxel, and birinapant. Birinapant appeared to increase NFκB reporter activity in the absence of TNFα, and this was attenuated by the decrease in Caspase 8. Birinapant did not block NFκB signaling in the presence of carboplatin or paclitaxel in either Caspase 8-high or Caspase 8-low cell lines. There was a trend toward decreased NFκB signaling in the NFκB-independent Ovcar8 cell line with the combination of birinapant, carboplatin, paclitaxel, and TNFα, perhaps suggesting that the presence of TNFα is a biomarker that should be included in future studies. The NFκB effects, however, were less informative than the apoptosis effects measured in this investigation.

Our studies highlighted that simultaneous administration of standard-of-care carboplatin, paclitaxel, and low concentrations of the SMAC-mimetic birinapant are synergistic with paclitaxel and effective in vitro and in vivo. Higher birinapant concentrations, however, were antagonistic to the carboplatin antitumor effect. The mechanism of SMAC-mimetic interference with carboplatin toxicity after simultaneous administration is not due to lack of Caspase 8 apoptotic activity but likely to an attenuation of carboplatin-induced DNA damage and the DNA repair response. Sequential administration of birinapant avoided the antagonistic effect. Taken together, carboplatin, paclitaxel, and birinapant combination treatments are synergistic and result in additional Caspase 8-independent tumor cell death and could benefit patient subpopulations who are resistant to apoptotic treatments. This work highlights the importance of understanding mechanisms of drug action in order to design optimal treatment schedules.

## Conclusions

Improved pathway-directed therapeutic advances are needed in order to improve ovarian cancer clinical outcome. Ovarian cancer populations exhibiting chemoresistance likely have defects in apoptotic signaling and circumvent treatments designed to induce tumor cell death by this mechanism. To enhance standard-of-care chemotherapy treatment of ovarian cancer, a tailored combination treatment of carboplatin, paclitaxel, and the SMAC-mimetic birinapant can enhance tumor cell death in vitro and in vivo, suggesting a useful, selective therapeutic benefit to specific patient subpopulations.

## Materials and methods

### Cell viability assays

Ovcar3 and Ovcar8 cells were previously described^4^ and represent PARP inhibitor-resistant ovarian cancer. All ovarian cell lines were authenticated by Short Tandem Repeat Analysis performed by the Molecular Detection Group, SAIC, Frederick National Laboratory, in reference to the ATCC profile for the same cell line (www.atcc.org). Adherent ovarian cancer cell viability was assessed using XTT (Sigma-Aldrich) as described^8^. Briefly, cells were seeded in 96-well plates at a density of 1–2000 cells/50 µl/well and incubated for 24 h. To analyze the lethal effects of cisplatin, carboplatin, paclitaxel, or birinapant on ovarian cancer cells, cells were Caspase 8 depleted using a previously validated short hairpin RNA (shRNA) clone^5^ or control scrambled shRNA. Cisplatin, carboplatin, and paclitaxel were from Tocris; birinapant was from Tetralogic, through a Materials Transfer Agreement^5^. All drugs were prepared as dimethyl sulfoxide (DMSO) stocks and serially diluted in RPMI medium to 2× concentrations, then added in 50 µl aliquots to each cell well. Final DMSO concentrations did not exceed 0.5%, previously established to be non-toxic to the cells. Plates were incubated for up to 10 days, and inhibitors replenished every 3–4 days. Cell viability was assessed by incubating cultures with XTT^8^ and absorbances read in a Spectramax M5 plate reader (Molecular Devices). Cell density in treated wells was expressed as a percentage of vehicle-treated control wells. Experiments included 3–6 replicate samples per point and were repeated at least three times. All viability studies were performed according to the above format, using 25 µM Caspase 8 inhibitor ZIETD (Z-IETD-FMK, FMK-007, R&D Systems), 20 µM NEC1 (Necrostatin-1, Tocris), or birinapant added to cells either alone or in combination, as described.

### Caspase activity assays

Caspase 8 and Caspase 3/7 activities were measured using cell cultures treated with chemotherapy, birinapant, or inhibitors for 18 h as described^5^. Activity data were normalized to viable cell number obtained by XTT assay, on duplicate assay plates. As in viability assays, experiments included 3–6 replicate samples per point and were repeated at least three times. Caspase 8 high (wild-type, WT) and Caspase 8 low (knockdown, KD) ovarian cancer cells were analyzed by cell-based Caspase 8 and Caspase 9 (G8200, G8090, G8210) luminescence assays (Promega), according to the manufacturer’s specifications.

### Patient datasets

Geo Dataset 9899 contains the gene expression for the AOCS samples^2^. Data were log 2 transformed prior to calculation. The TCGA data portal is found at https://tcga-data.nci.nih.gov/docs/publications/ov_2011/^3^. Expression data in the portal have been normalized to non-cancer controls. Gene expression data were grouped into designated subtypes, as per the respective publications, and the average expression of Caspase 8 and NFκB gene signature was calculated. The NFκB gene signature is previously published^4^. Kaplan–Meier survival plots were generated using online calculator at https://statcom.dk/K-M_plot.

### Xenograft experiments

Ovcar8 and Ovcar3 cell lines were obtained and maintained as described before^9^. Cells were authenticated via Short Tandem Repeat analysis by the Molecular Detection Group, Leidos, Frederick National Laboratory, in reference to the ATCC (www.atcc.org) and Cell Miner (http://discover.nci.nih.gov/cellminer) profile for the same cell line. Cells used for tumor induction were tested by MTBM as required by the NCI ACUC Committee and confirmed to contain no mouse viruses. Cells (1–2 × 10^6^) were counted and freshly prepared as suspensions in 0.5 ml phosphate-buffered saline for subcutaneous (flank) or intraperitoneal injections into 6–8-week-old athymic nu/nu female mice in randomly assigned groups of 10 mice. Drug treatments were administered intraperitoneally as described, freshly prepared in aqueous 0.5 M sodium citrate buffer pH 5.0. Mice were housed and observed according to approved NCI-ACUC guidelines. Total body weights and subcutaneous tumor caliper measurements were taken twice weekly in mice exhibiting palpable subcutaneous tumors until 5–6 weeks or humane endpoints. Subcutaneous tumor volumes were calculated according to the formula *V* = ½ (length × width^2^); tumor burden for mice injected intraperitoneally was assessed by monitoring body weight and histopathology analysis after 6–10 weeks or humane endpoints. Survival studies assessed mouse survival at experimental humane endpoints. Veterinary staff were blinded to treatment group. Statistical analysis of survival studies was done by log-rank test.

### Compusyn analysis

Analysis of dose effects after combination treatments of cells with carboplatin or paclitaxel with birinapant was performed using the CompuSyn for Drug Combinations and for General Dose-Effect Analysis software, as described^10^.

### NFκB activity reporter assays

Ovcar3 and Ovcar8 cells subjected to scrambled or Caspase 8 shRNA knockdown were first selected with puromycin as described before^4^. After selection, cells were transduced with a lentiviral vector containing an NFκB transcriptional regulatory element, using the Cignal Lenti Reporter System (CLS-013L), according to the manufacturer’s specifications and allowed 72 h for maximum vector expression. Transient reporter assays were subsequently performed for 18 h. Briefly, cells were plated in 96-well plates at a density of 10,000 cells/well. After overnight attachment, cells were exposed to serum starvation medium containing 0.5% fetal bovine serum for 24 h. Drugs or IKKβ inhibitor IV (EMD Biosciences) were added for 1 h after which TNFα (300-01A, PeproTech) was added to stimulate NFκB activity for 18 h. Control wells received vehicle alone. Luciferase activity was measured using the Luciferase Assay System (E4030, Promega) according to the manufacturer’s instructions and a Spectramax M5 plate reader (Molecular Devices). Luciferase units were normalized to viable cell number, obtained by XTT assay, on duplicate assay plates. Experiments included 3–6 replicate samples per point and were repeated at least three times.

### IL-8 mesoscale cytokine analysis

Ovcar8 cells expressing WT or low Caspase 8 levels were subjected to treatment with carboplatin, paclitaxel, birinapant, and NFκB inhibitor single or combined simultaneous treatments, for 18 h as described. Culture supernatants (100 μl) were collected after 18-h drug treatment and assessed for the levels of IL-8 using Mesoscale Kit (MSD K15025B) according to the manufacturer’s specifications. Experiments included eight replicate samples per cytokine and were repeated twice.

### p-γH2AX immunohistochemistry assay

Ovcar8 cells expressing WT or low Caspase 8 levels were subjected to treatment with carboplatin, birinapant, or combined simultaneous treatment with carboplatin and antagonistic dose of birinapant for 18 h. Cells were fixed with 4% paraformaldehyde and standard immunohistochemistry was performed to assess the levels of phosphorylated γH2AX as a measure of carboplatin-induced DNA damage. Subcutaneous xenograft tumors from mice treated with single, simultaneous, and sequential drug combinations were freshly fixed after a week of treatment and subjected to phosphorylated γH2AX level assessment using the same antibody (p-γH2AX antibody Millipore # 05-636-AF488). Experiment included ten mice per group.

### BOND-max Autostainer Staining/image capture and quantitation

The method used for staining formalin-fixed paraffin-embedded tissue sections was modified from our previously described Bond-max™ Autostainer Staining protocol^11^. The following antibody working solutions were prepared and loaded into the BOND-MAX Processing Module: 10 μg/ml monoclonal cleaved caspase-3 (R&D systems, MAB835, clone 269518, Milan, Italy) antibody prepared in Bond Primary Antibody Diluent (Leica Biosystems, Buffalo Grove, IL) and goat-anti-rabbit Alexa Fluor 546 (Invitrogen, Life Technologies, Grand Island, NY) antibody prepared 1:100 in 1× Bond Wash Solution; 5 µg/ml monoclonal anti-phospho (Ser 139) γH2AX-FITC conjugate (Millipore, 16-202a, clone JBW301, Billerica, MA) antibody was prepared in Bond Primary Antibody Diluent (Leica Biosystems, Buffalo Grove, IL); and DAPI dihydrochloride, FluoroPure™ grade (Invitrogen, Life Technologies, Grand Island, NY) prepared at 0.25 µg/ml in Bond Primary Antibody Diluent. Slides were cured overnight with Prolong Gold Antifade Reagent (Invitrogen) in the dark and imaged the following day. For long-term storage, slides were stored in the dark at −20 °C.

Slides stained with the cleaved caspase-3/γH2AX/DAPI multiplex and were scanned using the Aperio ScanScope FL image capture system (Leica Biosystems) for each xenograft or biopsy with at least six fields from each slide analyzed. Images were acquired using ×20 magnification with a Leica Plan Apo 20X/0.7NA objective, 0.4622 µm/pixel resolution, and images had a 16-bit depth. The cleaved caspase-3 exposure was 1.6 s, γH2AX exposure of 500 ms, and DAPI exposure 80 ms. Images were extracted with a fixed size (1000 × 1000 pixel) from full tissue scans using the Aperio ImageScope software. Images that were selected for extraction were derived from tumor tissue determined to be of sufficient quality as assessed during the evaluation of an adjacent hematoxylin and eosin–stained section of each specimen and necrotic regions of the tissue were avoided. Definiens Architect Tissue Studio IF software (Definiens AG, Munich, Germany) was used for quantitative analysis for γH2AX biomarker expression by percentage of γH2AX-positive nuclei analysis and nuclear segmentation and enumeration of all xenograft material. Additional analysis methods are described previously^11^.

### Western analysis

Total protein was extracted from described ovarian cell cultures expressing Caspase 8 or scrambled shRNA untreated, treated with chemotherapy, and/or birinapant using RIPA buffer (sc-24948) according to the manufacturer’s protocol (Santa Cruz Biotechnology, Santa Cruz, CA) and concentrations were estimated with the BCA Protein Assay Kit (Thermo Scientific, Rockford, IL). Sodium dodecyl sulfate-polyacrylamide gel electrophoresis and western blot analysis were performed using, respectively, the NuPage system (Invitrogen, Carlsbad, CA) and the Supersignal Chemiluminescent Substrate system (Thermo Scientific, Rockford, IL) using Phospho-ATM (Cell Signaling #4526), Phospho ATR (Abcam #ab227851), and GAPDH (Abcam #ab9485) antibodies.

### Statistical analysis

All in vitro experiments were performed a minimum of three independent experiments, with 3–8 technical replicates per experiment. Replicate numbers were maximized to ensure adequate power for assessing differences between groups. Statistical comparisons of significance were done by one-way analysis of variance with Tukey post hoc adjustment for multiple comparisons for viability, Caspase activity, and NFκB activity assays, as well as p-gH2.AX in vitro counts. Normal distribution and equal variance were assumed in the computations. Mouse survival curves were compared using Kaplan–Meier log-rank tests. With 10 mice per group, we sought at least 90% power to detect an effect size of 1.0 with alpha 0.1. Significance and *p* values comparisons are described in the figure legends.

## Supplementary information

Authorship form

Supplemental Figure 1

Supplemental Figure 2

Supplementary Figure Legends
