# Supplementary figures and images for: Smac-mimetic enhances antitumor effect of standard chemotherapy in ovarian cancer models via Caspase 8-independent mechanism
Source: Cell Death Discov. 2021 Jun 4;7:134. doi: 10.1038/s41420-021-00511-2 (PMC8178341; doi:10.1038/s41420-021-00511-2)

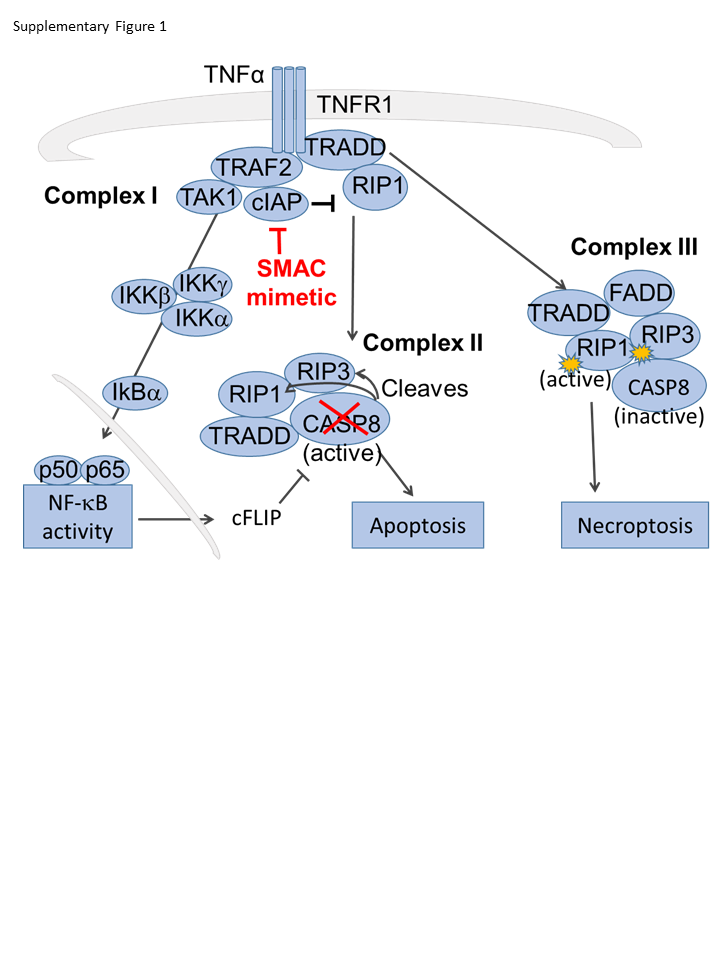

Supplement: Supplementary file 2 — Supplemental Figure 1 [file 41420_2021_511_MOESM2_ESM.tif]

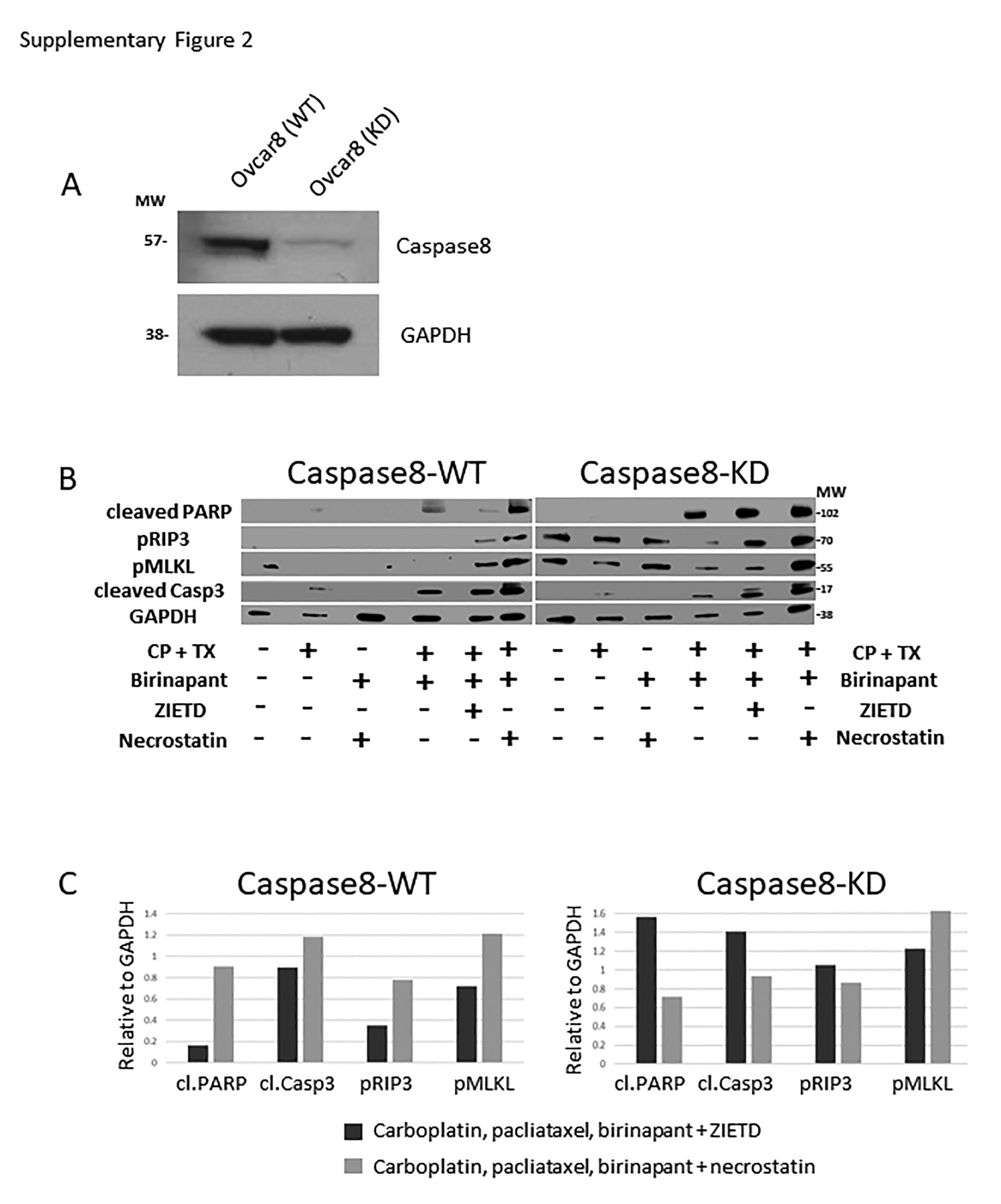

Supplement: Supplementary file 3 — Supplemental Figure 2 [file 41420_2021_511_MOESM3_ESM.tif]
