# Supplementary material for: Smac-mimetic enhances antitumor effect of standard chemotherapy in ovarian cancer models via Caspase 8-independent mechanism
Source: Cell Death Discov. 2021 Jun 4;7:134. doi: 10.1038/s41420-021-00511-2 (PMC8178341; doi:10.1038/s41420-021-00511-2)
Supplement: Supplementary file 4 — Supplementary Figure Legends [file 41420_2021_511_MOESM4_ESM.docx]

**Supplementary Figure 1.** Schematic representation of NFκB signaling downstream of TNFα stimulation, and regulation of apoptosis and necrosis by SMAC mimetic.

**Supplementary Figure 2.** **(A)** Western blot analysis of Caspase 8 protein expression in Ovcar8 cells transduced with scrambled (WT) or Caspase 8 (KD) shRNA. (B) Western blot of apoptosis and necroptosis markers under conditions as in Figure 1E. (C) quantification of proteins in B.
